# Effect of an educational intervention on the knowledge, attitudes, and practices of healthcare workers at King Hussein Cancer Center towards predatory publishers

**DOI:** 10.1186/s12909-023-04312-2

**Published:** 2023-05-22

**Authors:** Abdallah Al-Ani, Leen Al-Huneidy, Hala Sultan, Shahad Iqneibi, Jamil Nazzal, Asem Mansour, Maysa Al-Hussaini

**Affiliations:** 1 Office of Scientific Affairs and Research, King Hussein Cancer Center, Amman, Jordan; 2 School of Medicine, the University of Jordan, Amman, Jordan; 3 Department of Radiology, King Hussein Cancer Center, Amman, Jordan; 4 Department of Pathology and Laboratory Medicine, King Hussein Cancer Center, Amman, Jordan

**Keywords:** Predatory publishers, Oncology, Jordan, Educational intervention

## Abstract

**Aims:**

We explored the effect of an educational intervention on the knowledge, attitudes, and practices of healthcare workers (HCWs) towards predatory publishing.

**Methods:**

A retrospective pre-post quasi experimental design was implemented on HCWs within King Hussein Cancer Center (KHCC). Following a 60-min educational lecture, a self-administered questionnaire was completed by participants. Pre- and post-intervention scores for familiarity, knowledge, practices, and attitudes were compared using the paired sample t-test. Multivariate linear regression was used to identify predictors of mean differences (MD) of knowledge scores.

**Results:**

A total of 121 respondents completed the questionnaire. The majority of participants demonstrated underwhelming awareness of predatory publishing and average levels of knowledge of their characteristics. Furthermore, respondents did not take the necessary precautions to avoid predatory publishers. The intervention (i.e. the educational lecture) improved familiarity (MD: 13.4; 95%CI: 12.4 – 14.4; *p*-value < .001), knowledge of predatory journal’s characteristics (MD: 12.9; 95%CI: 11.1 – 14.8; *p*-value < .001), awareness and perceived compliance to preventive measures (MD: 7.7; 95%CI: 6.7 – 8.6; *p*-value < .001), and positively influenced attitudes towards open access and safe publishing (MD: 0.8; 95%CI: 0.2 – 1.5; *p*-value = 0.012). Females had significantly lower familiarity scores (*p*-value = 0.002). Moreover, those who had published in open access journals, received at least one predatory e-mail, or had more than 5 published original articles had significantly higher familiarity and knowledge scores (all *p*-value < 0.001).

**Conclusions:**

An educational lecture proved effective in improving awareness of KHCC’s HCW’s to predatory publishers. Nonetheless, the mediocrity of pre-intervention scores raises concerns on effectiveness of the predatory covert practices.

**Supplementary Information:**

The online version contains supplementary material available at 10.1186/s12909-023-04312-2.

## Introduction

The scientific landscape has dramatically changed with the advent of the World Wide Web. In response to unfair policies set by some publishers, open access (OA) publishing began to rise around the early 2000s [1]. The premise of OA is to provide peer-reviewed research as fast as it is published with little to no usage restrictions [2]. The OA movement has risen to ensure unrestricted access to research, eliminate intermediaries, and shift the cost of publication from readers onto authors [1]. This online medium of research distribution is characterized by shorter publication times, faster and broader dissemination of research, and more reliable access for scientists from developing nations [1, 3], all of which were changes welcomed in part by the academic and scientific communities [4].

Within academia, number of publications is used as a measure of academic efficacy. Moreover, publications and ratings (e.g., h-index) of researchers often dictate funding and are a central prerequisite for promotion within most universities and research centers [3, 5]. Thus, such incentives have led to increased pressure on researchers to publish, particularly those young and eager to hold a solid publication record early on in their careers [5, 6]. Considering the hostility of academia, elegantly described as “publish or perish”, and the shifting of publication costs towards authors, predatory journals exploited the OA model to derive monetary gain via article processing charges (APC) without meeting the minimum standards of quality assessment or peer review [1, 7, 8]. In 2014, over 10,000 predatory journals produced around 420,000 articles across a multitude of scientific disciplines rendering predatory publishing one of the greatest threats to scientific publishing since its early conception in 1665 [2, 5, 9].

Predatory journals promise extremely fast publication time, often within days or weeks, high acceptance rates, and report unverified impact factors [10]. One of the characteristics of these journals is the coverage of a wide range and oftentimes-unrelated disciplines. These journals that claim to cover a wide range of disciplines are characterized by unprofessional presentations and deceptive layouts that attempt to mimic upper echelon publishers [11].Young researchers who are often frustrated by a series of rejections and long publication times, might resort to publishing on predatory mediums [12]. However, they are ominous to the fact, mostly due to lack of experience, that such publications may ruin their reputation and demerit their legitimate publication record. While some predatory publishers slip into reputable indexes, many mediums of predatory publishing provide little to no indexing services, thus rendering their publications less accessible, less likely to be read and cited, and consequently useless in the scope of the greater body of scientific literature [10, 13, 14]. The predatory publishing market is estimated to be worth 74 million dollars [15]. Despite their aggressive and indiscriminate marketing, flexibility, and adaptability, the growing body of fraudulent publishers is rather fragile. Multiple sting operations demonstrated the unfathomable, or rather the lack of publishing standards of these publishers [6].

Only a handful of studies had explored the knowledge or awareness of predatory publishers and journals among healthcare workers. Moreover, evidence from Middle Eastern countries is even more scarce. Therefore, we investigated the effectiveness of an educational intervention on the knowledge, attitudes, and practice (KAP) of healthcare workers (HCWs) towards predatory publishing and journals. Such studies will enable us to predict the impact of such a phenomenon on the sanctity of scientific evidence produced within our region.

## Methodology

We implemented a retrospective pre-post quasi experimental design to assess the impact of an institutional educational intervention on the KAP of HCWs at King Hussein Cancer Center (KHCC) with regards to predatory publishers and journals. The retrospective pre-post design of assessing educational interventions has proved its validity and reliability across literature as it reflects the actual performance change that is statistically more accurate and of greater validity compared to its traditional counterpart [16]. Also, it reduces response-shift bias [17].

The implemented institutional educational intervention was a 60-min comprehensive lecture. In addition to the history and developments of academic publishing, the lecture contained the latest evidence on and definitions of predatory publishers stated in the 2019 Ottawa Consensus meeting [18]. The presented lecture showcased various exhibits of predatory publishers that were designated as such by Beall’s List, Cabell’s List, and the Directory of Open Access Journals (DOAJ). Participants were prompted to interactively spot predatory characteristics and discuss them with the lecturer. At various points within the intervention, participants were given the opportunity to discuss presented findings and reflect on previous experiences with predatory publishers to which they were oblivious at the time.

### Questionnaire development

Following the educational lecture, an online, self-administered questionnaire was completed by participants. We developed and validated a retrospective pre-post questionnaire to tackle the study’s aims. The tool was formulated after an extensive literature review tackling the topic across Europe and North America. The questionnaire is comprised of 6 domains: 1) demographics (4 items), 2) research background (6 items), 3) familiarly with predatory journals and OA publishing (6 items), 4) knowledge of predatory journals and publishers’ characteristics (15 items), 5) attitudes towards predatory publishing (8 items), and 6) practices pertaining to predatory publishing (6 items). Other variables and miscellaneous items are included with the attached questionnaire as supplementary material. Excluding demographics, the earlier 2 domains were mostly dichotomous (i.e., yes/no) or required continuous/numeric answers, while the latter were presented as 5-point Likert-scales.

The questionnaire’s content was approved by a panel of research experts within KHCC. Furthermore, pilot testing of the questionnaire demonstrated excellent internal consistency across all domains including familiarity (Cronbach α = 0.864), knowledge of predatory characteristics (Cronbach α = 0.913), practices towards safe publishing (Cronbach α = 0.878), and attitude domains (Cronbach α = 0.743). Meanwhile, face validity was ensured through respondents’ feedback during pilot testing. The results of factor analysis are provided as supplementary material (See Additional files 1 and 2).

### Sample size calculation

The estimated sample size was calculated using G*Power 3.1. At a power of 80%, α margin of error of 5% and an effect size of 30%, a sample of 90 participants was needed to demonstrate statistical differences of appropriate power when using paired sample t-test. Similarly, using the aforementioned parameters, a minimum of 120 participants was needed to demonstrate a statistically powerful difference using the paired-sample t-test.

### Statistical analysis

The data was analyzed using SPSS (IBM Corp, IBM SPSS Statistics for Windows, Version 23.0, Armonk, NY, USA). For items utilizing 5-point Likert scales, disagreement responses were grouped together, while agreement responses were grouped together for ease in reporting. Moreover, average 5-point Likert scales were reported as means ± standard deviations. Mean differences (MD) of pre- and post-interventional scores were evaluated using the paired-sample t-test and were reported along with their 95% confidence intervals (CI). Moreover, the aforementioned MDs were compared between various categorical groups using the student’s t-test and ANOVA. Total scores for the questionnaires’ domains (i.e., familiarity, knowledge of predatory characteristics, practices towards safe publishing, and attitudes towards predatory publishing) were calculated as the average mean of all items constituting said domain and compared between categories using the aforementioned statistical tests. A linear regression model was computed to explore the effectors on MD after the educational intervention. All statistical tests were conducted with a 95% CI and a 5% error margin. A *p*-value of less than 0.05 was considered statistically significant.

## Results

### Participants’ characteristics and research background

One hundred and twenty-one HCW’s at KHCC completed our survey. The overall response rate was 73.3%. The studied sample comprised of 35.5% males and 64.5% females, with an average age of 37.4 ± 9.3 years. The most common respondents were nurses (28.9%), clinical pharmacists (27.3%), followed by senior oncology consultants (19.0%). Median years of experience within respective fields was 11.0 (5.0, 16.50) years. In terms of research background, most respondents published 5 or less original manuscripts in their careers (68.6%), published in an OA journal (55.4%), and received a predatory email at least once (64.5%). Table 1 demonstrates the characteristics of respondents.

**Table 1:** Characteristics of recruited respondents (*n* = 121)

Variable	*n* (%)
**Sex**	
Male	43 (35.5)
Female	78 (64.5)
**Work Designation**	
Intern	3 (2.5)
Resident	1 (0.8)
Junior consultant	6 (5.0)
Senior consultant	23 (19.0)
Nurse	35 (28.9)
Pharmacist	33 (27.3)
Medical student	2 (1.7)
Others	18 (14.9)
**Published manuscripts throughout one’s career**	
≤ 5	83 (68.6)
6 – 10	15 (12.4)
11 – 15	6 (5.0)
16 – 20	2 (1.7)
21 – 25	4 (3.3)
26 – 30	2 (1.7)
> 30	9 (7.4)
**Published manuscripts as first author**	
≤ 5	100 (82.6)
6 – 10	10 (8.3)
11 – 15	4 (3.3)
16 – 20	3 (2.5)
21 – 25	2 (1.7)
26 – 30	0 (0.0)
> 30	2 (1.7)
**Published manuscripts as corresponding author**	
≤ 5	99 (81.8)
6 – 10	13 (10.7)
11 – 15	3 (2.5)
16 – 20	2 (1.7)
21 – 25	1 (0.8)
26 – 30	0 (0.0)
> 30	3 (2.5)
**Publishing in Open Access journals**	
Yes	67 (55.4)
No	54 (44.6)
**Receiving predatory emails**	
Yes	78 (64.5)
No	43 (35.5)

### Impact of educational intervention

In terms of familiarity with predatory publishing, the majority of participants demonstrated an underwhelming rate of awareness. Participants declared lack of familiarity with OA publishing mechanics, the existence of predatory publishers, preventive measures (i.e., Think, Check, and Submit), and were unable to identify a predatory journal. Similarly, average responses on knowledge of predatory publishers’ characteristics did not portray the necessary level of expected confidence (Refer to Supplementary table 1; Additional file 1). Our educational intervention significantly improved both participants’ familiarity and knowledge of predatory publishers across all items (all *p*-value < 0.001).

With respect to followed practices to ensure safe publishing, average scores demonstrated that KHCC researchers did not take the necessary precautions to ensure the validity of a journal such as using the Clarivate Journal Citation Report, matching the journal’s credentials with the DOAJ, or verifying the journal’s editorial board (Refer to Supplementary table 2; Additional file 1). The implemented educational intervention demonstrated significant improvements to awareness and perceived future compliance to preventive measures against predatory publishing across all items (all *p*-value < 0.001).

In terms of attitude, participants were aware of the benefits of OA publishing and had a slight preference towards OA than subscription-based journals (Refer to Supplementary table 3; Additional file 1). Moreover, they demonstrated a distaste with regards to aggressive predatory e-mails and the impact of predatory publications on the integrity of the scientific literature. Furthermore, KHCC participants denounced that predatory publishing could be justified even in circumstances of resource scarcity. Our educational intervention significantly improved the attitudes of participants towards OA in terms of overall preference (*p*-value = 0.001), appreciation of its increased accessibility (*p*-value = 0.009), and awareness of its positive effects on citations (*p*-value < 0.001).

**Fig. 1:**
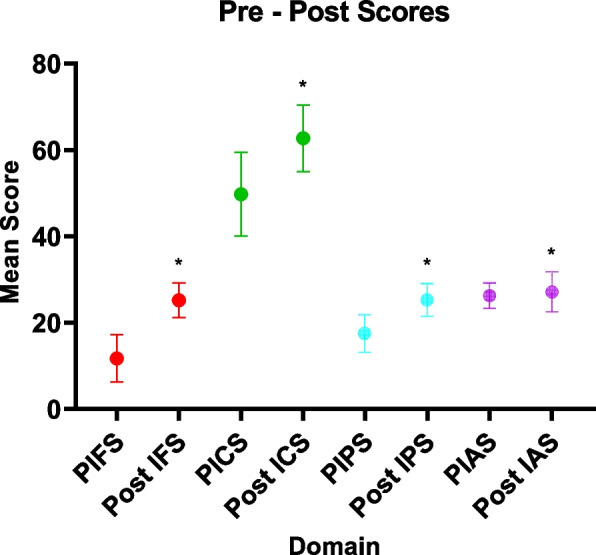
Effect of educational intervention of calculated scores. PIFS: Pre-intervention familiarity score, Post IFS: Post-intervention familiarity score, PICS: Pre-intervention characteristics score, Post ICS: Post-intervention characteristics score, PIPS: Pre-intervention practices score, Post IPS: Post-intervention practices score, PIAS: Pre-intervention attitudes score, Post IAS: Post-intervention attitudes score. “*” Denotes statistical significance at *p*-value < 0.05

When creating composite scores for all four tested subscales (i.e., familiarity, knowledge of predatory characteristics, preventive practices, and attitudes), our implemented educational intervention was able to significantly improve scores for all four subscales (Refer to Fig. 1). The intervention improved familiarity (MD: 13.4; 95%CI: 12.4 – 14.4; *p*-value < 0.001), knowledge of predatory journals’ characteristics (MD: 12.9; 95%CI: 11.1 – 14.8; *p*-value < 0.001), awareness and perceived compliance to preventive measures (MD: 7.7; 95%CI: 6.7 – 8.6; *p*-value < 0.001), and positively influenced attitudes towards OA and safe publishing (MD: 0.8; 95%CI: 0.2 – 1.5; *p*-value = 0.012) (refer to Table 2).

**Table 2:** Pre- and post-interventional scores

	Pre-educational intervention (Mean ± SD)	Post-educational intervention (Mean ± SD)	Mean Difference (95%CI)	Lower 95% CI	Upper 95% CI	*p*-value
Familiarity score	11.7 ± 5.5	25.2 ± 4.0	13.4	12.4	14.4	** < .001**
Characteristics/knowledge score	49.7 ± 9.7	62.6 ± 7.7	12.9	11.1	14.8	** < .001**
Practices score	17.5 ± 4.3	25.3 ± 3.7	7.7	6.7	8.6	** < .001**
Attitudes score	26.3 ± 2.9	27.1 ± 4.6	0.8	0.2	1.5	**0.012**

### Factors affecting pre-intervention scores

Univariate analysis demonstrated that females had significantly lower familiarity scores (*p*-value = 0.002). Moreover, those who had published in OA journals, received at least one predatory e-mail in their lifetime, or had more than 5 published original articles had significantly higher familiarity scores (all *p*-value < 0.001). Similarly, respondents who fall within those aforementioned three groups had significantly higher levels of knowledge of predatory journals’ characteristics (all *p*-value < 0.001). Interestingly, participants that had published in OA journal or received a predatory email had significantly better attitude scores than their counterparts (*p*-value = 0.025 and 0.004, respectively). Table 3 demonstrates factors affecting both pre- and post-intervention scores. Finally, it appears that age demonstrated a significantly weak yet positive correlation with both familiarity (*r* = 0.275; *p*-value = 0.002) and knowledge scores (*r* = 0.207; *p*-value = 0.023). On the other hand, years of experience within respective fields did not correlate with any score.

**Table 3:** Associations between calculated scores and participants’ characteristics

	Familiarity score		Characteristics score		Practices score		Attitudes score	
	Pre-intervention	Post-intervention	Pre-intervention	Post-intervention	Pre-intervention	Post-intervention	Pre-intervention	Post-intervention
**Sex**								
Male (*n* = 43)	13.7 ± 5.7	25.4 ± 4.2	49.0 ± 10.0	61.2 ± 9.2	17.4 ± 4.7	24.7 ± 3.7	25.7 ± 2.6	26.4 ± 5.0
Female (*n* = 78)	10.6 ± 5.0	25.0 ± 3.9	50.1 ± 9.5	62.9 ± 6.9	17.6 ± 4.1	25.6 ± 3.7	26.5 ± 2.9	27.5 ± 4.3
* p*-value	**0.002**	0.554	0.574	0.589	0.831	0.188	0.126	0.224
**Publishing status**								
Non-OA publications (*n* = 54)	9.7 ± 4.1	24.9 ± 4.1	46.3 ± 9.7	63.8 ± 7.5	16.8 ± 4.3	26.1 ± 3.6	25.6 ± 2.6	26.4 ± 4.4
Published in OA (*n* = 67)	13.3 ± 5.9	25.3 ± 3.9	52.4 ± 8.7	61.7 ± 7.8	18.1 ± 4.2	24.6 ± 3.8	26.8 ± 2.9	27.8 ± 4.7
* p*-value	** < .001**	0.574	** < .001**	0.142	0.105	**0.032**	**0.025**	0.124
**Number of publications**								
< 5 publications (*n* = 83)	9.9 ± 4.2	25.6 ± 4.3	47.6 ± 9.9	62.7 ± 7.7	17.2 ± 4.1	25.7 ± 3.7	25.9 ± 2.7	26.6 ± 4.3
> 5 publications (*n* = 38)	15.6 ± 5.8	26.4 ± 2.7	54.2 ± 7.4	62.6 ± 7.9	18.3 ± 4.7	24.3 ± 3.6	26.9 ± 3.1	28.3 ± 4.8
* p*-value	** < .001**	**0.017**	** < .001**	0.945	0.188	**0.049**	0.106	0.062
**Aggressive solicitation**								
Received predatory e-mails (*n* = 78)	13.2 ± 5.9	26.3 ± 3.5	52.8 ± 8.4	62.9 ± 8.0	17.9 ± 4.4	25.4 ± 3.8	26.8 ± 3.2	27.8 ± 4.8
Did not receive predatory e-mails (*n* = 43)	9.1 ± 3.6	23.2 ± 4.1	44.1 ± 9.5	62.2 ± 7.3	16.9 ± 4.1	25.1 ± 3.6	25.2 ± 1.9	25.7 ± 4.0
* p*-value	** < .001**	** < .001**	** < .001**	0.641	0.189	0.648	**0.004**	**0.017**

### Multivariate analysis

Linear regression demonstrates that pre-interventional familiarity score (B: -0.403; 95%CI: -0.797 – -0.010; *p*-value = 0.045) and practice score (B: -0.861; 95%CI: -1.253 – -0.468; *p*-value < 0.001) were negative predictors of MD in characteristics score. Participants who were more familiar with OA publishing and predatory journals and those with positive attitudes towards safe OA publishing demonstrated significantly lower MD in characteristic scores after being subjected to the educational intervention (Refer to Table 4).

**Table 4:** Predictors of mean differences in characteristics score

	**Mean differences in characteristics score**			
	**Linear regression model**			
	**B**	**Lower 95% CI for (B)**	**Upper 95% CI for (B)**	***p*-value**
Age	-0.305	-0.610	0.000	0.050
Sex (Female)	-1.844	-5.403	1.714	0.307
Years of experience	0.157	-0.142	0.455	0.300
Published in OA	-4.365	-7.961	-0.769	**0.018**
Received predatory emails	-2.766	-6.604	1.072	0.156
Pre-Intervention Familiarity Score	-0.403	-0.797	-0.010	**0.045**
Pre-Intervention Practice Score	-0.861	-1.253	-0.468	**0.000**
Pre-Intervention Attitudes Score	0.017	-0.553	0.587	0.953
Frequently incorporate the literature into clinical decisions	-0.476	-3.721	2.768	0.772
Number of publications	0.411	-0.818	1.639	0.509

## Discussion

In this study, we demonstrated that HCWs, working at the Middle East’s most specialized cancer center, showed an underwhelming awareness towards the existence of predatory journals, were unable to identify most of their characteristics, and did not utilize precautionary measures to avoid them when publishing. Moreover, we highlighted that a single well-organized lecture is effective in improving the familiarity, knowledge, practices, and attitudes of HCWs towards predatory publishers. Finally, while females had lower familiarity scores, those who had published in Open Access journals had at least one experience to predatory solicitation, or had more than 5 published original articles, had significantly higher familiarity and knowledge scores.

Our earlier results are strongly echoed within the available yet scant literature. The current body of literature demonstrates that the impending hazards of predatory publishers are largely unknown to medical practitioners across a variety of disciplines. Castro-Martinez et al. [19] showed that 83.6% of practitioners within medicine and social sciences were not aware of predatory businesses. Richtig et al. [20] showed that 29.4% of Australian dermatologists were familiar with predatory journals, while only 11.9% declared that they were able to identify them. Similarly, Maurer et al. [21] demonstrated that 39.9% of German orthopedic and trauma surgeons were familiar with predatory journals when only 29.6% and 21.0% were familiar with the “*think, check and submit”* approach and the DOAJ, respectively. In addition, Richtig et al. [22] illustrated that 69.7% of oncologists from various societies (i.e., Austrian Association of Haematology and Oncology; Working Group Medial Oncology Within German Cancer Association; and German Society of Haematology and Oncology) had prior knowledge of predatory journals, but only 54.8% were able to identify such journals. Finally, a survey among prospective veterinary and medical authors from various institutions reported that the awareness of predatory journals, the DOAJ, and Beall’s list was prevalent among only 23.0%, 4.8%, and 23.9% of participants [23]. Among our participants, only 43.6% were familiar with the phenomenon of predatory publishing while only 36.9% reported being able to identify such journals.

Similar to the general lack of knowledge of predatory journals and publications among medical practitioners, as mentioned in the previous paragraph, the rates of awareness towards predatory publishers display an extreme variance among other professionals including students and faculty members. Studies demonstrate extremely low rates of predatory literacy among Jordanian, Saudi, and New Zealand medical undergraduates ranging from 7.0% to 9.0% [24, 25]. On the other hand, 70.5% of faculty members at the Oakland University had heard of predatory publishers and 60.0% could correctly identify them across their field of expertise [26]. When reporting on the perspectives of authors publishing in predatory journals, Cohen et al., [27] demonstrated that the greater majority of authors publishing in predatory journals are alarmingly uninformed about the nature of such businesses. The study also showed that editors were significantly more familiar with predatory practices compared to authors, a difference that was consistent with awareness of Beall’s list too, thus ability to identify predatory publishers. On a similar note, Cobey et al. [28] showed that among authors who had published in predatory biomedical journals, only 3.9% were aware that they were submitting to a predatory publisher.

It appears that the awareness of such a phenomenon is not related to resource scarcity, nor sex of the researcher but is often associated with research activity. Across multiple reports, factors such as workplace environment, number of publications, high-impact publications, previous experiences with predatory publishers/OA, or recent scientific activity, were shown to be significantly associated with higher knowledge of predatory journals [22, 26]; a finding consistent with our results. Interestingly, we found that females had significantly less familiarity scores than their male counterparts. This is mostly attributed to meaningful differences in ability to identify predatory journals as male HCWs were significantly more confident in reporting identification familiarity than their female counterparts. Nonetheless, both males and females had comparable rates in terms of awareness towards the existence of predatory journals.

Overall, across our results and that of the published international literature, a significant portion of medical practitioners lacked the ability to recognize the presence of such an epidemic, let alone its characteristics. The issue with recognizing predatory publishers is that their characteristics may not always overlap and at times may contradict each other. Moreover, there exists more than 90 checklists to identify fake publishers, only four of which are evidence-based. Thus, on April of 2019 at Ottawa, Canada, scholars representing various academic societies established the most prominent characteristics of predatory journals including: false or misleading information, deviation from best editorial and publications practices, lack of transparency, and aggressive, indiscriminate solicitation [18].

Our findings have a plethora of important implications. First, young or inexperienced researchers require guidance from professionals within the field. Such researchers often misunderstand the nature of predatory journals and are often unaware of the long term consequences on their reputation, and on the quality of evidence provided to readers accessing such publications [23]. Second, our findings imply that researchers, even those working in a workplace that thrives on medical research and consistently produces publications across various disciplines of oncology such as KHCC, can be oblivious to the presence and growth of predatory publishers. This particular phenomenon only showcases the efficiency and effectiveness of the adaptability and covert practices exercised by those publishers, or maybe it hints at the futility of academia’s solutions in tackling predatory publishers [29]. Third, as highlighted by Richtig et al. [22], a broad educational intervention must be designed in order to help researchers identify predatory publishers.

Our study, in terms of design and purpose, falls in line with the aforementioned recommendations others made throughout the literature [30]. We demonstrated that a 60-min lecture, designed to holistically tackle the concept of predatory journals, is extremely potent as it significantly improves the knowledge, attitudes, and practices of researchers towards predatory publishers. Moreover, it provides inexperienced researchers with the necessary exposure to consider the existence of such a phenomenon in the first place. However, the most important attribute of this intervention is it being a cost-effective and simple method that institutions can easily adopt while also providing a significant impact. Interventions, such as those implemented in this study, do not only provide a superficial outlook on predatory publishers, but also a framework for augmenting researchers’ scholarly publishing literacy. Such a concept is defined as the ability to differentiate authentic from counterfeit journals as to publish their research output through the most appropriate outlet in their field [31]. For scholarly publishing literacy to advance and thrive within today’s academic landscape, a researcher must develop a deep understanding of the operations, policies, and implications of OA publishing so that they exercise diligent checking of journals using an empirical criteria [32]. This can be achieved through librarian-oriented re-skilling of researchers through providing information literacy, increasing accessibility of tools and databases, and promoting digital scholarships.

Among our participants, preventive practices for safe publishing were as underwhelmingly displayed as levels of knowledge. Prior to the implemented intervention, participants demonstrated that they consistently did not check the credentials of journals they publish in. Such credentials include impact factor, ISSN, editorial board, and memberships, among others. This recklessness may originate from two sources: a general lack of awareness consistent with the literature or a general sense of safety conveyed by KHCC’s research policies. Researchers at KHCC are encouraged to publish in high impact journals and are often only recognized for publications made in highly ranking journals and/or reputable publishers. Nonetheless, despite the myriad of practices that can be implemented to avoid predatory journals, these journals display an adaptive and slippery presence within highly regarded indexes. The credibility of curated indexes or databases, such as Web of Science, DOAJ, PubMed, Scopus, is now being questioned as multiple predatory publishers have creeped into their logs. Multiple reports demonstrate that questionable publishers were found within trustworthy research references (e.g., MEDLINE, EMBASE, Scopus) and citation repositories (e.g., SCIE and ESCI), the latter of which are already plagued with poor quality manuscripts published in deceptive journals [8, 33, 34]. Reports show that, for instance, a predatory publisher may reside within the archives of PubMed Central without being qualified for the inclusion within the MEDLINE database [8]. Such discrepancy rises due to having two different review committees for each database with different regulations/standards. While not extremely prevalent, the mere existence of predatory publishers within such reputable databases may pose extreme danger to the practice of conducting secondary research (i.e., meta-analysis) which utilizes databases as its core data mining mechanic [35]. It seems that despite the high level of control within major databases, indexing is not really synonymous with quality anymore [9].

The propagation of predatory journals have led to a distortion of the published scientific literature [10]. A handful of studies have demonstrated the presence of a significant number of predatory publishers across a variety of medical disciplines including neuroscience, urology, emergency medicine, orthopedics, rehabilitations, anesthesiology, and pathology among many others [33, 36–41]. The publication of “scientific” work that undergoes dubiously fast peer review under little to zero quality assurance standards incentivizes the production of fake, plagiarized, and fraudulent work; thereby undermining the value of scientific literature [8]. These practices promote the propagation of errors as the existence of low-quality, unethical, or fabricated work might set the baseline for other studies citing such materials and further disseminating untrustworthy facts. Thus, an alternate science is produced through the grafting of many papers, often self-citing, which sought to explore an observation that was never measured rigorously in the first place. These questionable results may resurface as references in articles published in legitimate scientific journals [14, 42].

While these journals provide their articles for free, their detrimental impact on patient education, physician decision making, and evidence synthesis is unknown [10, 35]. Thus, the distribution of questionable articles within predatory mediums has the potential to impact public health for decades if enough misinformation is spread, similar to that of the aftermath of the Wakefield case [43]. Predatory publishers, due to a complete lack of regulation, do not invest any resources in order to uncover hidden conflicts of interest thus assisting in the spread of intentionally biased results [44]. Finally, since the primary motive of predatory publishers is monetary gain, the moment their model becomes cost-ineffective, those responsible would rather pull the plug on the entire website than maintain its infrastructure at a loss [3]. This results in the loss of all published content including those that offer legitimate and valuable results.

Nevertheless, despite our efforts to tackle the subject of predictor journals, our findings are subject to a few limitations. While the study recruited HCWs from Jordan’s largest cancer institution and one of the Middle East’s most advanced cancer centers, the results may not be generalized to the entirety of the region thus setting a geographical limitation. Researches dependent on questionnaires face the issue of recall and social desirability biases which may underreport negative findings. Moreover, due to the design of the study, participants may be motivated to portray subject bias as an attempt to visualize improvement in their skills or knowledge. Finally, a longitudinal assessment of projected practices cannot be implemented but is the subject of a later publication.

## Conclusion

In short, we demonstrated that HCWs at the KHCC had subpar levels of knowledge and awareness with regards to predatory publishers. Moreover, participants demonstrated a lack of diligent checking of journals at time of submission. However, we proved the efficiency of a cost-effective educational intervention in the improvement of participants KAP towards this serious epidemic that is plaguing all medical disciplines. The current body of literature is merely concerned with investigating the characteristics of authors publishing in predatory journals or the awareness of researchers towards such phenomenon. Thus, more research is needed to examine the efficacy of anti-predatory recommendations and solutions proposed in various review papers and commentaries.

## Supplementary Information

**Additional file 1.**

**Additional file 2.**

## Data Availability

The datasets used and/or analyzed during the current study are available from the corresponding author on reasonable request.
