# Supplementary material for: Effect of an educational intervention on the knowledge, attitudes, and practices of healthcare workers at King Hussein Cancer Center towards predatory publishers
Source: BMC Med Educ. 2023 May 22;23:355. doi: 10.1186/s12909-023-04312-2 (PMC10201791; doi:10.1186/s12909-023-04312-2)
Supplement: Supplementary file 1 — Additional file 1. [file 12909_2023_4312_MOESM1_ESM.pdf]

Data collection instrument

Domain	Name	Answer Key
**Domain I**	**Demographics**	
	Age (in years)	Continuous
	Gender/Sex	Categorical (e.g., male)
	Work designation	Categorical (e.g., resident)
	Years of experience within respective field	Continuous
**Domain II**	**Research background**	
	Do you incorporate the scientific literature you read in your therapeutic/diagnostic decisions?	5-point Likert scale
	Approximately, how many manuscripts (e.g., original articles) have you published throughout your career?	Categorical (e.g., 6 – 10)
	Approximately, how many manuscripts (e.g., original articles) have you published throughout your career as a First Author?	Categorical (e.g., 6 – 10)
	Approximately, how many manuscripts (e.g., original articles) have you published throughout your career as a Corresponding Author?	Categorical (e.g., 6 – 10)
	Have you published anything in an Open Access (OA) journal?	Dichotomous (e.g., yes, no)
	Do you receive e-mail invitation from journals wanting you to contribute to an article?	Dichotomous (e.g., yes, no)
**Domain III**	**Familiarity [Were you familiar with…]**	
	Open Access (OA) publishing?	5-point Likert scale
	The phenomenon of predatory publishing?	5-point Likert scale
	"Beall's List"?	5-point Likert scale
	The "Directory of Open Access Journals" (DOAJ)?	5-point Likert scale
	The [Think, Check, and Submit] approach before publishing?	5-point Likert scale
	How to identify a predatory journal?	5-point Likert scale
**Domain IV**	**Knowledge [Do you believe that…]**	
	"No Impact Factor" is a characteristic related to predatory practices	5-point Likert scale
	"Promotion of metrics such as Index Copernicus, CiteFactor" is a characteristic related to predatory practices	5-point Likert scale
	"Poor to no indexing" is a characteristic related to predatory practices	5-point Likert scale
	"Provide Open Access to all articles" is a characteristic related to predatory practices	5-point Likert scale
	"Low quality of published articles' content" is a characteristic related to predatory practices	5-point Likert scale
	"Low quality of published articles' presentation (e.g., grammatical or spelling errors)" is a characteristic related to predatory practices	5-point Likert scale
	"No or extremely hasty peer review" is a characteristic related to predatory practices	5-point Likert scale
	"Lack of a reliable editorial board" is a characteristic related to predatory practices	5-point Likert scale
	"Journal is located in a developing country (e.g., Nigeria, India, etc)" is a characteristic related to predatory practices	5-point Likert scale
	"Unprofessional journal website layout" is a characteristic related to predatory practices	5-point Likert scale
	"Low publication fees" is a characteristic related to predatory practices	5-point Likert scale
	"Rapid publication process" is a characteristic related to predatory practices	5-point Likert scale
	"No submission system (i.e., submission through e-mail)" is a characteristic related to predatory practices	5-point Likert scale
	"Misleading journal titles (e.g., World Journal of Pediatric Science)" is a characteristic related to predatory practices	5-point Likert scale
	"Aggressively e-mailing practitioners for submissions" is a characteristic related to predatory practices	5-point Likert scale

**Domain V**	**Practices [When choosing a journal…]**	
	I use the Clarivate Journal Citation Report (JCR) for guidance	5-point Likert scale
	I check the journal's credentials via the DOAJ	5-point Likert scale
	I check the journal's credentials via its membership in COPE (Committee on Publication Ethics)	5-point Likert scale
	I verify the journal's editorial board	5-point Likert scale
	I check for details pertaining to peer review, editorial policies, or article-processing fees, thus ensuring transparency	5-point Likert scale
	I consult my institution's librarian for guidance	5-point Likert scale
**Domain VI**	**Attitudes**	
	Publication cost is my number one and only concern when choosing a journal	5-point Likert scale
	I prefer publishing in Open Access rather than a subscription-based journal	5-point Likert scale
	I don't mind publishing in journals interested in recruiting me via email	5-point Likert scale
	Open Access publishing makes my scientific contributions more accessible	5-point Likert scale
	Open Access publishing makes my scientific contributions more cited	5-point Likert scale
	Low-cost publishing in low quality journals is justified considering the resource scarcity in Jordan	5-point Likert scale
	Low-cost publishing in low quality journals has no effect on the integrity of scientific literature within a field	5-point Likert scale
	Publishers characterized by low cost, fast peer review, and fast publication process provide a suitable alternative to the bigger greedy journals, particularly for young researchers	5-point Likert scale

Supplementary table 1: Pre and post interventional scores per each questionnaire item (Domain III and IV)

	Pre-educational intervention (Mean ± SD)	Post-educational intervention (Mean ± SD)	Mean Difference	Lower 95% CI	Upper 95% CI	p-value*
**Familiarity [Were you familiar with…]**						
Open Access (OA) publishing?	2.7 ± 1.4	4.2 ± 0.9	-1.5	-1.7	-1.3	**< .001**
The phenomenon of predatory publishing?	2.1 ± 1.3	4.3 ± 0.8	-2.2	-2.4	-2.0	**< .001**
"Beall's List"?	1.3 ± 0.8	4.1 ± 0.9	-2.7	-2.9	-2.5	**< .001**
The "Directory of Open Access Journals" (DOAJ)?	1.8 ± 1.1	4.1 ± 0.8	-2.3	-2.5	-2.1	**< .001**
The [Think, Check, and Submit] approach before publishing?	1.8 ± 1.1	4.3 ± 0.8	-2.5	-2.7	-2.3	**< .001**
How to identify a predatory journal?	1.9 ± 1.2	4.1 ± 0.8	-2.2	-2.4	-1.9	**< .001**
**Knowledge [Do you believe that…]**						
"No Impact Factor" is a characteristic related to predatory practices	3.1 ± 1.0	3.8 ± 1.2	-0.67	-0.93	-0.41	**< .001**
"Promotion of metrics such as Index Copernicus, CiteFactor" is a characteristic related to predatory practices	2.9 ± 0.9	3.8 ± 1.0	-0.79	-1.01	-0.57	**< .001**
"Poor to no indexing" is a characteristic related to predatory practices	3.3 ± 1.0	4.0 ± 1.0	-0.69	-0.88	-0.49	**< .001**
"Provide Open Access to all articles" is a characteristic related to predatory practices	2.9 ± 1.0	3.5 ± 1.2	-0.66	-0.87	-0.44	**< .001**
"Low quality of published articles' content" is a characteristic related to predatory practices	3.6 ± 0.9	4.4 ± 0.9	-0.75	-0.94	-0.56	**< .001**
"Low quality of published articles' presentation (e.g., grammatical or spelling errors)" is a characteristic related to predatory practices	3.6 ± 0.9	4.5 ± 0.7	-0.88	-1.04	-0.72	**< .001**
"No or extremely hasty peer review" is a characteristic related to predatory practices	3.5 ± 0.9	4.3 ± 0.8	-0.85	-1.02	-0.67	**< .001**
"Lack of a reliable editorial board" is a characteristic related to predatory practices	3.5 ± 0.9	4.4 ± 0.9	-0.82	-1.01	-0.63	**< .001**

"Journal is located in a developing country (e.g., Nigeria, India, etc)" is a characteristic related to predatory practices	3.1 ± 0.9	4.0 ± 0.9	-0.92	-1.10	-0.74	**< .001**
"Unprofessional journal website layout" is a characteristic related to predatory practices	3.6 ± 0.9	4.5 ± 0.6	-0.87	-1.03	-0.71	**< .001**
"Low publication fees" is a characteristic related to predatory practices	2.9 ± 0.9	3.8 ± 1.1	-0.94	-1.13	-0.75	**< .001**
"Rapid publication process" is a characteristic related to predatory practices	3.3 ± 0.9	4.4 ± 0.7	-1.12	-1.30	-0.94	**< .001**
"No submission system (i.e., submission through e-mail)" is a characteristic related to predatory practices	3.4 ± 0.9	4.4 ± 0.8	-0.96	-1.14	-0.79	**< .001**
"Misleading journal titles (e.g., World Journal of Pediatric Science)" is a characteristic related to predatory practices	3.3 ± 9.6	4.4 ± 0.8	-1.20	-1.37	-0.99	**< .001**
"Aggressively e-mailing practitioners for submissions" is a characteristic related to predatory practices	3.5 ± 0.9	4.3 ± 0.9	-0.82	-1.00	-0.63	**< .001**

*Mean differences were analyzed using the paired-sample t-test

Supplementary table 2: Pre and post interventional scores per each questionnaire item (Domain V)

	Pre-educational intervention (Mean ± SD)	Post-educational intervention (Mean ± SD)	Mean Difference	Lower 95% CI	Upper 95% CI	p-value*
**Practices [When choosing a journal…]**						
I use the Clarivate Journal Citation Report (JCR) for guidance	2.8 ± 0.8	4.0 ± 0.8	-1.21	-1.40	-1.01	**< .001**
I check the journal's credentials via the DOAJ	2.8 ± 0.9	4.2 ± 0.8	-1.38	-1.57	-1.18	**< .001**
I check the journal's credentials via its membership in COPE (Committee on Publication Ethics)	2.8 ± 0.9	4.2 ± 0.8	-1.31	-1.51	-1.09	**< .001**
I verify the journal's editorial board	2.9 ± 0.9	4.3 ± 0.7	-1.33	-1.52	-1.13	**< .001**
I check for details pertaining to peer review, editorial policies, or article-processing fees, thus ensuring transparency	3.1 ± 0.9	4.3 ± 0.7	-1.21	-1.41	-1.02	**< .001**
I consult my institution's librarian for guidance	3.0 ± 0.9	4.3 ± 0.8	-1.26	-1.44	-1.08	**< .001**
*Mean differences were analyzed using the paired-sample t-test						

Supplementary table 3: Pre and post interventional scores per each questionnaire item (Domain VI)

	Pre-educational intervention (Mean ± SD)	Post-educational intervention (Mean ± SD)	Mean Difference	Lower 95% CI	Upper 95% CI	p-value*
**Attitudes**						
Publication cost is my number one and only concern when choosing a journal	2.7 ± 0.9	2.7 ± 1.3	-0.02	-0.24	0.19	0.821
I prefer publishing in Open Access rather than a subscription-based journal	3.2 ± 0.8	3.5 ± 1.1	-0.34	-0.55	-0.14	**0.001**
I don't mind publishing in journals interested in recruiting me via email	2.7 ± 0.8	2.6 ± 1.3	1.98	-0.04	0.44	0.114
Open Access publishing makes my scientific contributions more accessible	3.6 ± 0.9	3.9 ± 1.0	-0.28	-0.50	-0.07	**0.009**
Open Access publishing makes my scientific contributions more cited	3.5 ± 0.9	3.9 ± 0.9	-0.40	-0.60	-0.20	**< .001**
Low-cost publishing in low quality journals is justified considering the resource scarcity in Jordan	2.9 ± 0.9	3.0 ± 1.3	-0.13	-0.35	0.08	0.235
Low-cost publishing in low quality journals has no effect on the integrity of scientific literature within a field	2.6 ± 1.0	2.9 ± 1.4	-0.26	-0.47	-0.05	**0.016**
Publishers characterized by low cost, fast peer review, and fast publication process provide a suitable alternative to the bigger greedy journals, particularly for young researchers	3.0 ± 0.9	2.9 ± 1.4	0.02	-0.21	0.26	0.839
*Mean differences were analyzed using the paired-sample t-test						

Factor Analysis

Construct validity was examined using factor analysis. Barret test for sphericity was significant at $P<0.001$ and Kaiser Meyer Olkin measure of 0.865. Using principal component analysis with direct oblimin rotation, a total of 6 factors had eigenvalues greater than one and explained 67.3% of the variance. Scree plot inspection demonstrated the point of inflexion after 4 components which explained 57.7% of the variance. The other components were excluded as they managed to explain less than 5% of the variance. The four components corresponded to the familiarity, knowledge of characteristics, attitudes, and practice domains [Refer to **Supplementary Figure 1; additional file 2**].

Supplementary Figure 1: Model representation of the familiarity, knowledge of characteristics, attitudes, and practices domains through factor analysis represented by factor loadings.
