# Supplementary figures and images for: Effect of an educational intervention on the knowledge, attitudes, and practices of healthcare workers at King Hussein Cancer Center towards predatory publishers
Source: BMC Med Educ. 2023 May 22;23:355. doi: 10.1186/s12909-023-04312-2 (PMC10201791; doi:10.1186/s12909-023-04312-2)

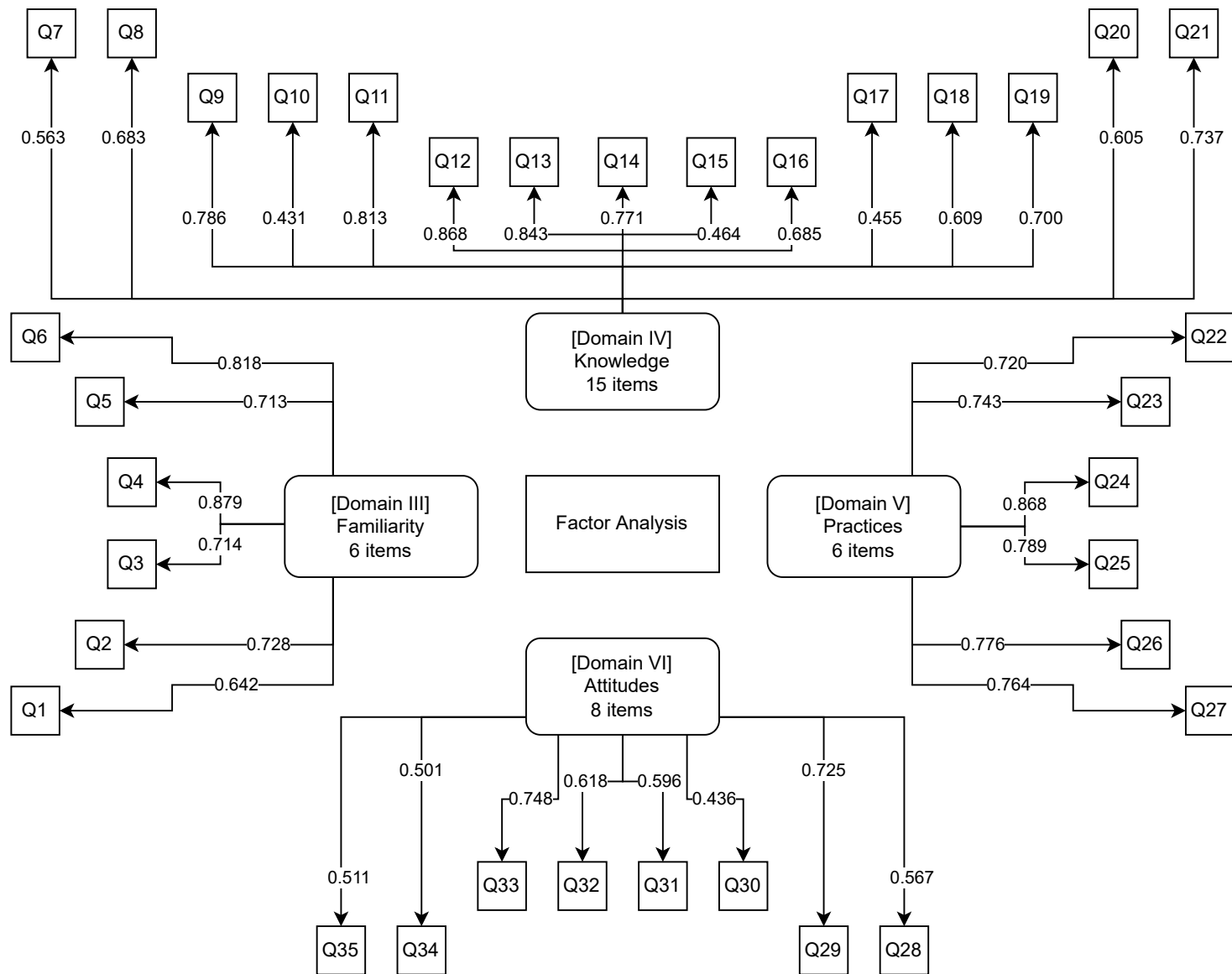

Supplement: Supplementary file 2 — Additional file 2. [file 12909_2023_4312_MOESM2_ESM.pdf]
